# Organocatalyzed enantioselective desymmetrization of aziridines and epoxides

**DOI:** 10.3762/bjoc.9.192

**Published:** 2013-08-15

**Authors:** Ping-An Wang

**Affiliations:** 1 Department of Medicinal Chemistry, School of Pharmacy, Fourth Military Medical University, Changle Xilu 17, Xi-An, 710032, P. R. China

**Keywords:** aziridine, desymmetrization, enantioselectivity, epoxide, organocatalysis

## Abstract

Enantioselective desymmetrization of *meso*-aziridines and *meso*-epoxides with various nucleophiles by organocatalysis has emerged as a cutting-edge approach in recent years. This review summarizes the origin and recent developments of enantioselective desymmetrization of *meso*-aziridines and *meso*-epoxides in the presence of organocatalysts.

## Introduction

**Figure 1:**
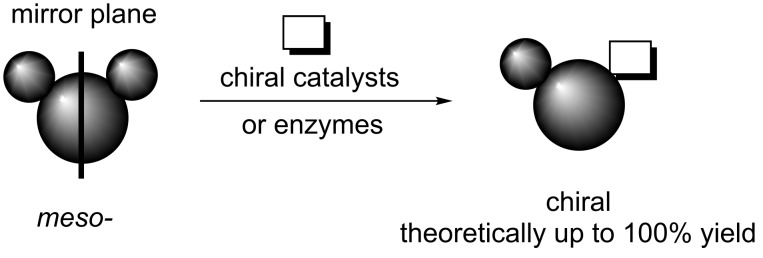
The catalyzed enantioselective desymmetrization.

The high demand of enantiopure organic compounds in the fine chemical industry gives impetus to the development of chiral technologies. Among them, the catalytic asymmetric synthesis represents the state of the art in organic chemistry [1]. Over the past four decades, the asymmetric synthesis based on transition-metal catalyzed reactions has given rise to a variety of significant achievements both in academic and industrial chemistry [2–3]. Desymmetrization is the modification of a molecule which results in the loss of symmetry elements such as a mirror plane, a center of inversion or a rotation–reflection axis (Figure 1). Usually, a prochiral or *meso*-molecule can be converted into a chiral molecule in a single step [4–6] in the presence of chiral catalysts. Therefore, enantioselective desymmetrization is regarded as a very powerful strategy for producing a large amount of chiral compounds from readily available achiral substrates. The past decade witnessed the renaissance and the golden age of organocatalyzed reactions [7–9]. Aziridines and epoxides are quite reactive due to the large tension of their three-membered ring system. The enantioselective ring-opening of aziridines [10–12] or epoxides [13–15] provides facile access to various chiral amines and alcohols and their derivatives with two adjacent stereogenic centers, which are widely used as building blocks in pharmaceutical and organic synthesis. The enantioselective catalytic desymmetrization of *meso*-aziridines and *meso*-epoxides by metal-based Lewis acids cooperating with chiral ligands has been well established [16–28]. However, the enantioselective desymmetrization of *meso*-aziridines and *meso*-epoxides catalyzed by small chiral organic molecules has only emerged in recent years. The goal of this review is to give a comprehensive overview on newly developed strategies in the field of organocatalyzed enantioselective desymmetrization of *meso-*aziridines and *meso*-epoxides.

## Review

### Organocatalyzed enantioselective desymmetrization of *meso*-aziridines

Many examples of the ring-opening of aziridines by various carbon [29–30], nitrogen [31–32], oxygen [33–34], sulfur [35–36] and halogen [37–39] nucleophilic reagents in the presence of catalytic amounts of small acids and bases such as BF_3_·Et_2_O, AcOH, TsOH, TFA, PBu_3_, TMEDA, TBAF, Bu_4_NHSO_4_, pyridine *N*-oxide, and *N*-heterocyclic carbenes were published. However, the enantioselective ring-opening of *meso*-aziridines in the presence of chiral organocatalysts (**OC**) has emerged as a research field only in recent years. The organocatalysts utilized in these processes are diverse in their structural features including cinchona alkaloid derivatives, chiral phosphoric acids, chiral amino alcohols, chiral thioureas, chiral guanidines, and chiral 1,2,3-triazolium chlorides. In this review, the research work of enantioselective desymmetrization of *meso*-aziridines is organized into sections according to the employed organocatalysts.

#### Cinchona alkaloid derivatives

**Figure 2:**
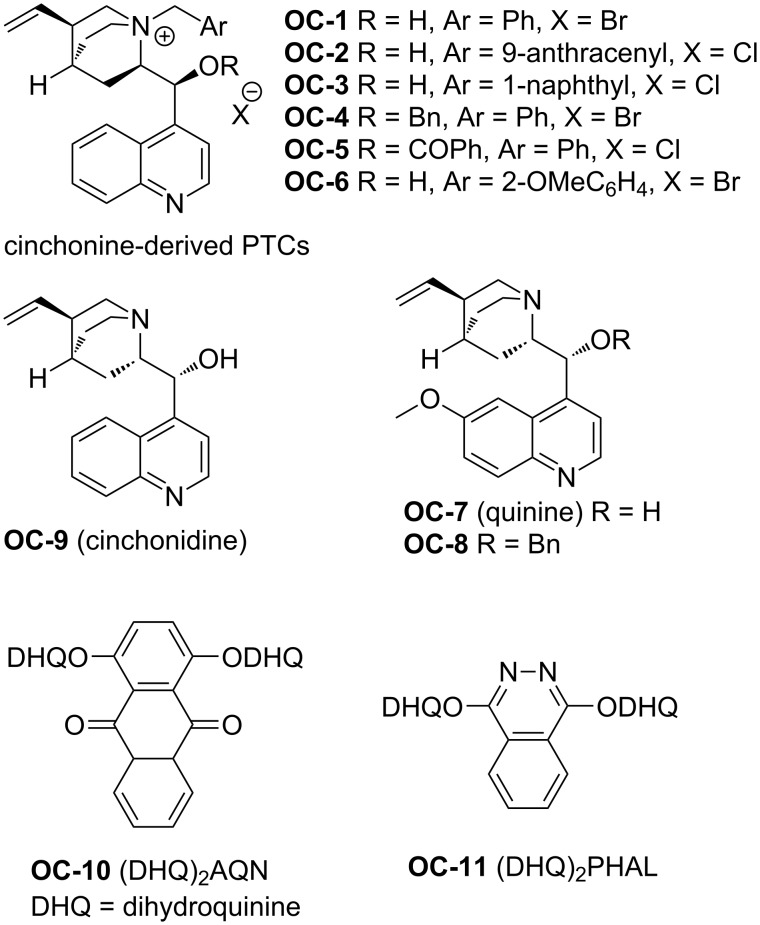
Cinchona alkaloid-derived catalysts **OC-1** to **OC-11**.

**Scheme 1:**
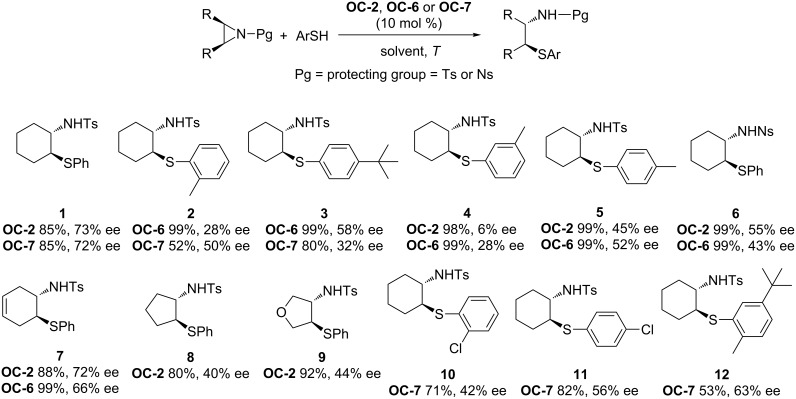
The enantioselective desymmetrization of *meso*-aziridines in the presence of selected Cinchona alkaloid-derived catalysts.

The first organocatalytic enantioselective desymmetrization of *meso*-aziridines was discovered by Hou and co-workers in 2007 [40] with various arylthiols as nucleophiles in CCl_4_ at 0 °C in the presence of cinchonine-derived phase-transfer catalysts (PTCs, Figure 2, **OC-1** to **OC-6**). The substituent on the bridgehead nitrogen of cinchona alkaloids has a great impact on the enantioselectivity of the reactions. The catalyst **OC-2** with 9-anthracenylmethyl on the bridgehead nitrogen is more efficient than other cinchona alkaloid-derived catalysts for the desymmetrization of *meso*-*N*-tosylaziridine (tosyl = Ts) as it affords the corresponding chiral *N*-Ts thioamines (Scheme 1, **1** to **9**,) in high yield (85%) and satisfactory enantioselectivity (73% ee). The **OC-4** and **OC-5** with a protecting group on 9-OH decreased the enantioselectivity dramatically. In the presence of **OC-6**, when *p*-nitrophenylsulfonylaziridine (Ns-aziridine) was employed as substrate, the corresponding *N*-Ns thioamine **8** was obtained in quantitative yield but with moderate enantioselectivity (55% ee). The need of two equivalents (equiv) of CsOH·H_2_O as a base in the procedure limited its application in organic synthesis.

Wu and co-workers have established the enantioselective ring-opening of *meso*-aziridines with arylthiols by using various quinine derivatives (**OC-7** to **OC-11**) as organocatalysts (Scheme 1) [41]. It was found that the desymmetrization performed well in CHCl_3_ in the presence of 10 mol % of quinine (**OC-7**) at room temperature. The β-amino sulfides were obtained in high yields (up to 85%) and with moderate enantioselectivities (up to 72% ee). However, **OC-10** and **OC-11** afforded extremely disappointing results for desymmetrization and the corresponding β-amino sulfide was obtained nearly racemic (3% ee). The usage of Boc for the *N*-protected group in aziridine resulted in no enantioselectivity under the optimum conditions.

**Figure 3:**
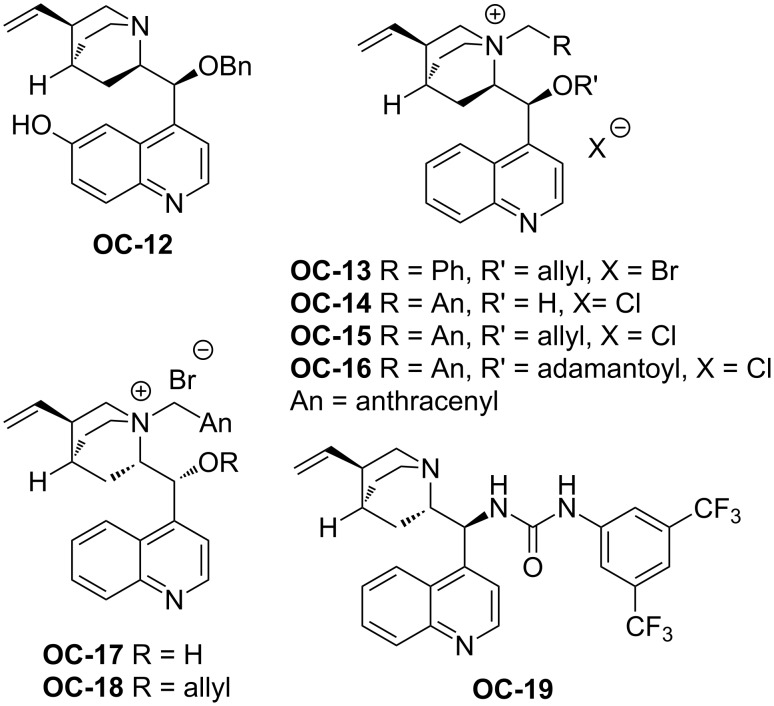
Cinchona alkaloid-derived catalysts **OC-12** to **OC-19**.

**Scheme 2:**
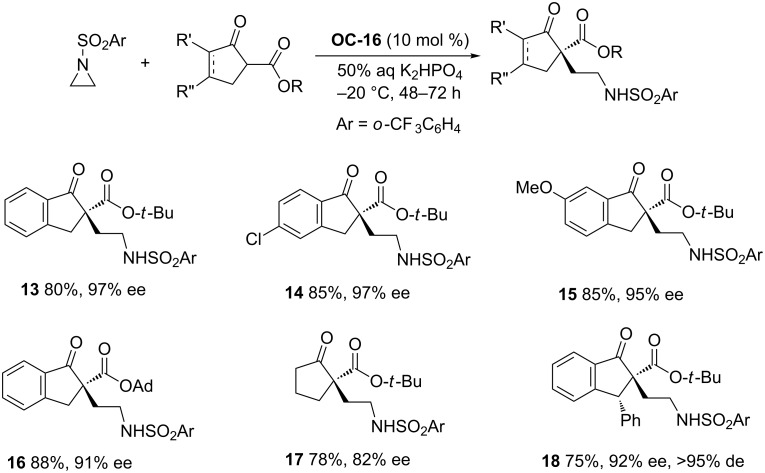
The enantioselective ring-opening of aziridines in the presence of **OC-16**.

The organocatalytic enantioselective ring-opening of *N*-protected aziridines by β-ketoesters by means of cinchona alkaloid-derived PTCs (Figure 3, **OC-12** to **OC-19**) for producing γ-amino acid derivatives with one stereogenic quaternary carbon has been independently developed by Dixon [42] and Jørgensen’s group [43]. Dixon and co-workers discovered that **OC-16** bearing bulky substituents both on the 9-*O* atom and the bridgehead nitrogen afforded excellent enantioselectivity of desirable products (Scheme 2, **13** to **18**, up to 97% ee) with 50% aqueous K_2_HPO_4_ as base. However, under the same conditions, the PTCs with a free 9-OH (**OC-17**) led to **13** in low yields and enantioselectivities (21% ee). The urea catalyst **OC-19** proved to be very inefficient and afforded **13** in 10% yield with 6% ee.

**Scheme 3:**
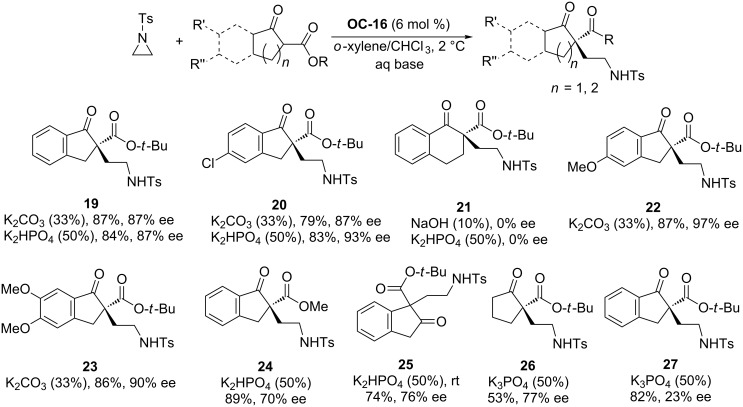
**OC-16** catalyzed enantioselective ring-opening of aziridines.

By shifting the *N*-protecting group in aziridines from *o*-(trifluoromethyl)benzenesulfonyl to Ts, Jørgensen and colleagues found that excellent enantioselectivities (up to 97% ee) of the ring-opening products (Scheme 3, **19** to **27**) were obtained by using **OC-16** as a catalyst, 33 wt % aqueous K_2_CO_3_ as a base and a low reaction temperature of −20 °C. This afforded perfect enantioselectivity (99% ee) of compound **19**, but at the sacrifice of the chemical yield (decrease to 21%). The *N*-Boc and *N*-Cbz protected *meso*-aziridines yielded the product in trace amounts under the optimum conditions.

#### Chiral phosphoric acids

**Figure 4:**
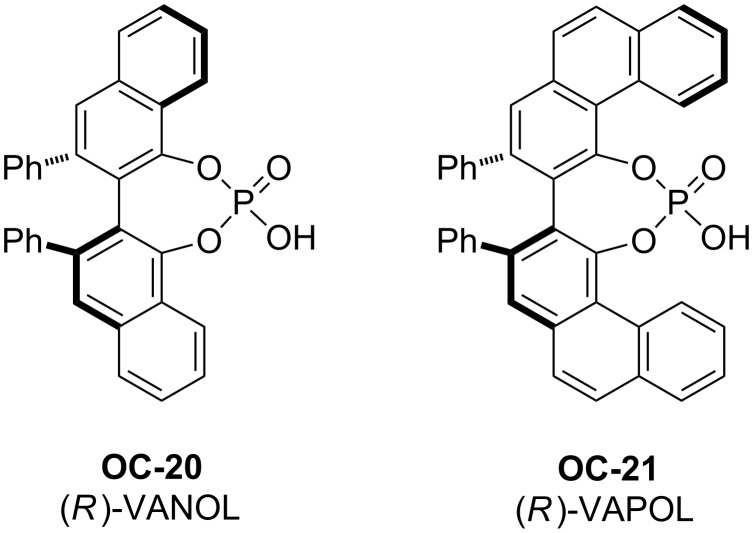
The chiral phosphoric acids catalysts **OC-20** and **OC-21**.

**Scheme 4:**
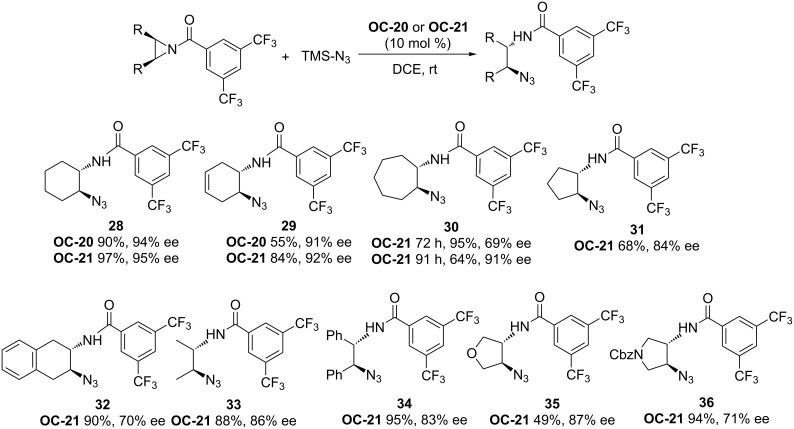
**OC-20** and **OC-21** catalyzed enantioselective desymmetrization of *meso*-aziridines.

**Figure 5:**
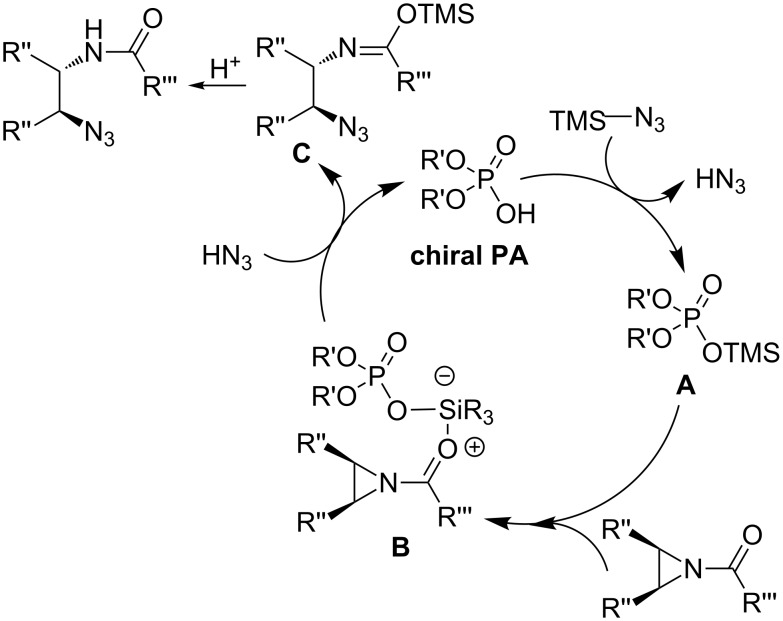
The proposed mechanism for chiral phosphorous acid-induced enantioselctive desymmetrization of *meso*-aziridines (**chiral PA** = **OC-21**).

An elegant enantioselective desymmetrization of *meso-*aziridines catalyzed by chiral phosphoric acids **OC-20** and **OC-21** as catalysts was carried out by Antilla [44] and co-workers (Figure 4). When a bis(3,5-trifluoromethyl)benzoyl group was used for the *N*-activation of *meso*-aziridines, 1,2-azidoamides (Scheme 4, **28** to **36**) were obtained in excellent yields (up to 97%) and enantioselectivities (up to 95% ee) by using TMSN_3_ as a nucleophile in the presence of 10 mol % of **OC-20** or **OC-21** in 1,2-dichloroethane (DCE) at room temperature. Interestingly, tetrabutylammonium azide or NaN_3_ were unreactive as a nucleophile under the same conditions, so the presence of the trimethylsilyl group is crucial to this ring-opening reaction. A proposed mechanism was based on NMR spectroscopic investigations as depicted in Figure 5. In the first step of the reaction, the active catalyst was formed by the displacement of the azide to produce HN_3_ and the chiral silane **A**. The latter activates the aziridine by means of coordinating to the carbonyl functionality of the aziridine to afford intermediate **B**. Species **B** was then attacked by HN_3_, resulting in **C** and the release of phosphoric acid. Compound **C** can be readily decomposed on silica gel to form the final product. Antilla and co-workers have also shown the enantioselective desymmetrization of *meso*-aziridines with functionalized mercaptans in the presence of (*S*)-VAPOL in ether at rt to afford β-(*N*-acylamino)phenyl thioethers in good yields and ee’s [45].

**Scheme 5:**
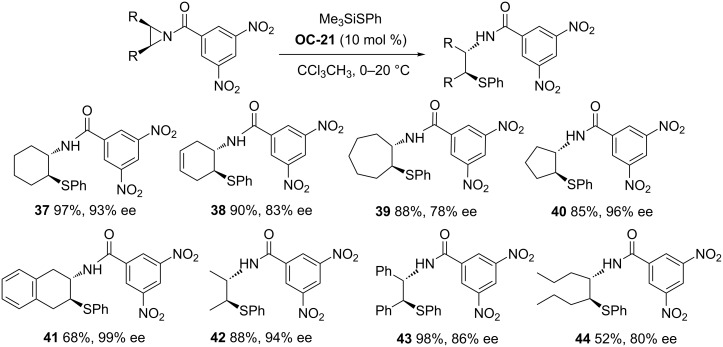
**OC-21** catalyzed enantioselective desymmetrization of *meso*-aziridines by Me_3_SiSPh.

By using 10 mol % of commercially available chiral phosphoric acid **OC**-**21** as a catalyst, Lattanzi [46] and colleagues have developed a facile desymmetrization of *meso*-*N*-acylaziridines with Me_3_SiSPh to produce β-(*N*-acylamino)phenyl thioethers (Scheme 5, **37** to **44**) in high enantioselectivities (78–99% ee). Interestingly, the aziridines with a six-membered ring were desymmetrized to give the corresponding thioethers in excellent yields and enantioselectivities when the molar ratio of aziridine/nucleophile/catalyst is 1.0:1.5:0.1. For the unreactive acyclic and seven-membered ring aziridines, the good enantioselectivities were obtained under the reverse ratio of the reagents.

**Scheme 6:**
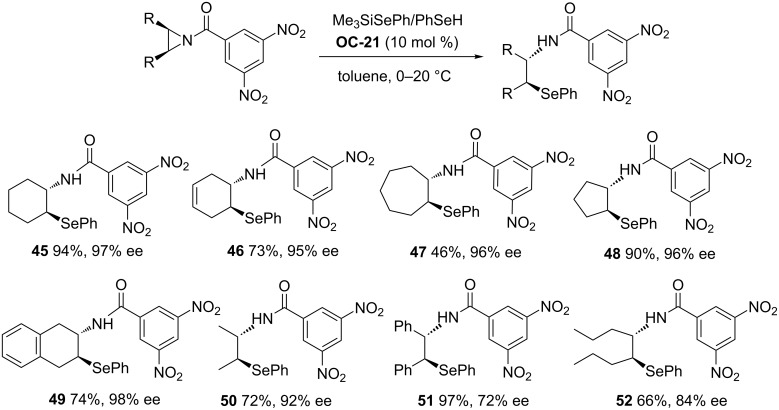
**OC-21** catalyzed the enantioselective desymmetrization of *meso*-aziridines by Me_3_SiSePh/PhSeH.

Della Sala’s group [47] has demonstrated the first example of a *meso*-aziridine desymmetrization with selenium nucleophiles to produce chiral β-aminoselenides in high enantioselectivities (up to 99% ee). Surprisingly, when the sterically hindered air-stable selenosilane *t*-BuMe_2_SiSePh was used as a sole nucleophile for desymmetrization of *meso*-*N*-acylaziridines in the presence of 10 mol % of chiral phosphoric acid **OC-21**, the good conversions and enantioselectivities were accomplished in toluene but with a very long reaction time (usually up to 4 days). The desymmetrization of *meso*-*N*-acylaziridines by the less sterically hindered selenosilane Me_3_SiSePh under the same conditions can be completed in several hours with good yields but moderate ee’s. According to the proposed mechanism, the formation of a small amount of PhSeH was involved in the induction step of the catalytic cycle and it acted as the actual nucleophile for the desymmetrization. In such a way a modified protocol based on the use of a combination of Me_3_SiSePh and PhseH as a nucleophile was developed. This modification proved to be very valuable and the desymmetrization can be accomplished within several hours to afford the chiral β-aminoselenides (Scheme 6, **45** to **52**) in both high yields (up to 97%) and enantioselectivities (up to 97% ee).

Recently, Della Sala [48] has re-examined the enantioselective desymmetrization of *meso*-aziridines with various silylated reagents (Me_3_SiN_3_, Me_3_SiSMe, Me_3_SiSPh, Me_3_SiSBn, Me_3_SiNCS and Me_3_SiSePh/PhSeH) by chiral phosphoric acid **OC-21**. It was found that both purchased and synthetic **OC-21** exhibited high catalytic activities and enantioselective inductions in the desymmetrization of *meso*-aziridines. Samples of **OC-21** were washed with aq HCl to generate metal-free chiral phosphoric acid **OC-21**, which was reused as the catalyst, and the corresponding ring-opening products were obtained in moderate yields as racemates. By using a 1:1 mixture of calcium and magnesium phosphate salts of **OC-21** as the catalyst, the corresponding ring-opening product **28** was produced in 94% yield and 91% ee. Therefore, calcium and magnesium phosphate salts of **OC-21** proved to be the true catalysts in these processes. The author suggested that these metal phosphates may be generated through the purification of **OC-21** on silica gel column. A dual Lewis-base activation mechanism of this desymmetrization was proposed.

#### Chiral amino alcohols

**Figure 6:**
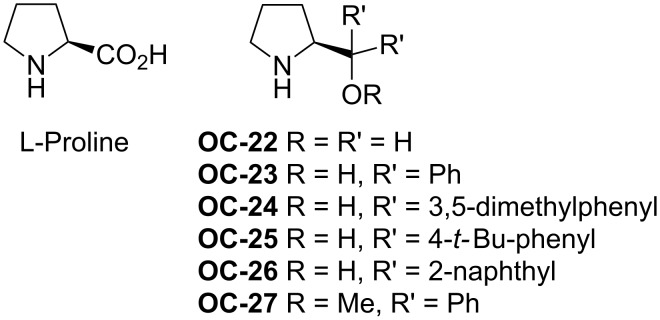
*L*-Proline and its derivatives **OC-22** to **OC-27**.

**Scheme 7:**
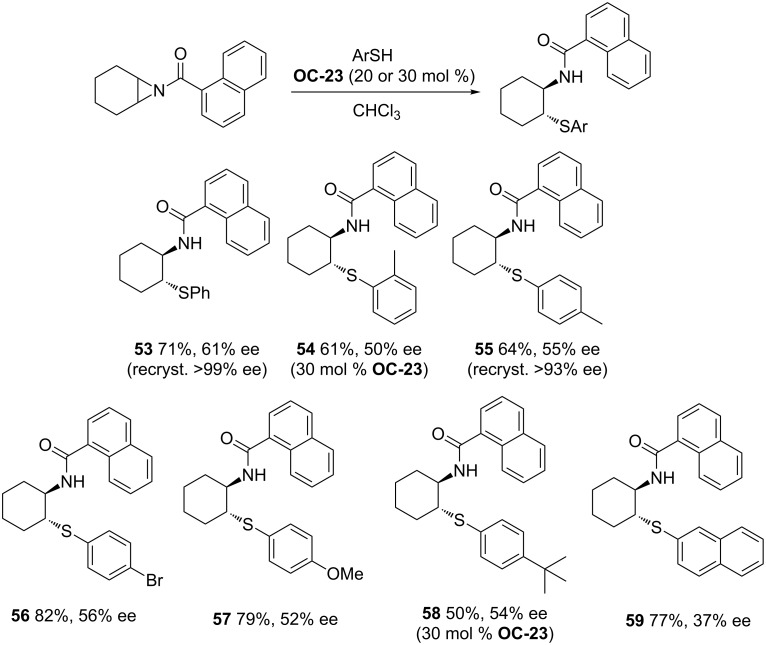
**OC-23** catalyzed enantioselective desymmetrization of *meso*-aziridines.

**Figure 7:**
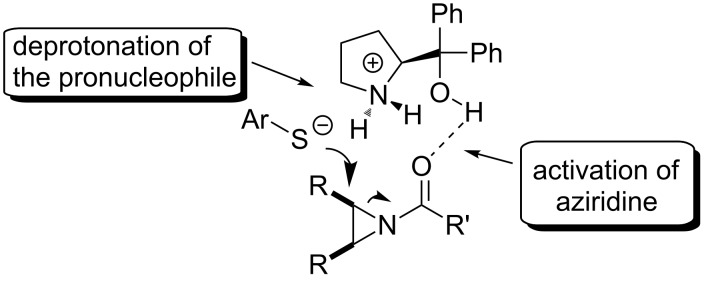
Proposed bifunctional mode of action of **OC-23**.

Lattanzi and colleagues [49] have also discovered the desymmetrization of *meso*-*N*-acylaziridines with benzenethiols in the presence of α,α-diaryl-*L*-prolinols (Figure 6, **OC-22** to **OC-25**) to afford the products in good yields and moderate enantioselectivities (up to 61% ee, Scheme 7). The dual activation mode of α,α-diphenyl-*L*-prolinol (**OC-23**) for the desymmetrization of *meso*-*N*-acylaziridines is proposed in this context (Figure 7). The arylthiol was deprotonated by the pyrrolidinyl-N of the organocatalyst to produce a highly active nucleophile, while the *meso*-*N*-acylaziridine was activated by the tertiary-hydroxy group of the organocatalyst by hydrogen-bonding interaction with the carbonyl-O of the acyl group in *meso*-*N*-acylaziridine. The latter was attacked by the highly active thio anion to furnish chiral β-amino thioethers in good yields. Interestingly, *meso*-*N*-tosylaziridines reacted sluggishly in both chloroform and toluene as the solvents under the same catalytic system, and the reason of this phenomenon is unknown.

#### Chiral thioureas

**Figure 8:**
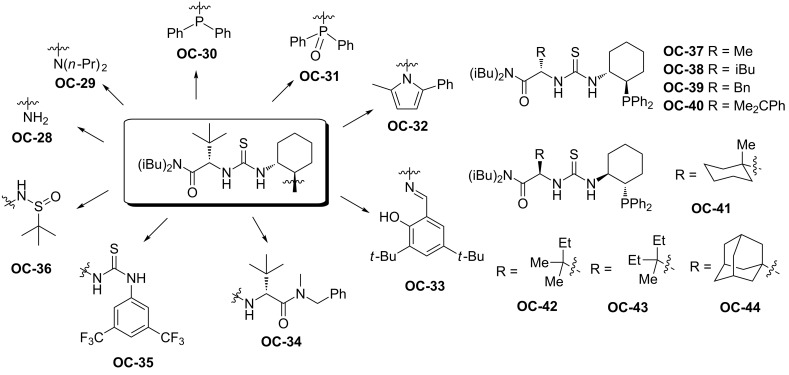
The chiral thioureas **OC-28** to **OC-44** for the desymmetrization of *meso*-aziridines.

**Scheme 8:**
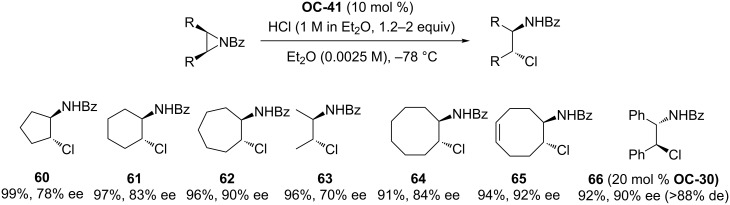
Desymmetrization of *meso*-aziridines with **OC-41**.

Jacobsen [50] and co-workers presented an enantioselective catalytic desymmetrization of *meso*-*N*-benzoylaziridines with HCl by a series of chiral thioureas (Figure 8, **OC-28** to **OC-44**). In most of the cases, a diluted reaction mixture is necessary to obtain good enantioselectivities and **OC-41** proved to be the best catalyst to provide β-chlorobenzamides (Scheme 8, **60** to **66**) in high yields and enantioselectivities (up to 92% ee). The ^31^P NMR studies of the interaction between phosphinothiourea **OC-41** and HCl indicated that the initial heterolysis of HCl affords a phosphonium chloride complex which is involved in the catalytic cycle. The desymmetrization of both cyclic and acyclic aziridines proceeded smoothly to give the corresponding β-chlorobenzamides in high yields and enantioselectivities.

#### Chiral guanidines

**Figure 9:**
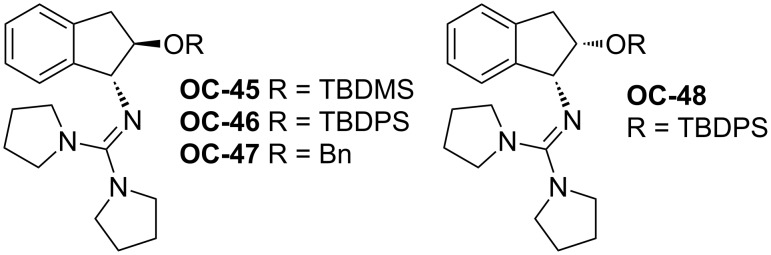
The chiral guanidines (**OC-45** to **OC-48**).

**Scheme 9:**
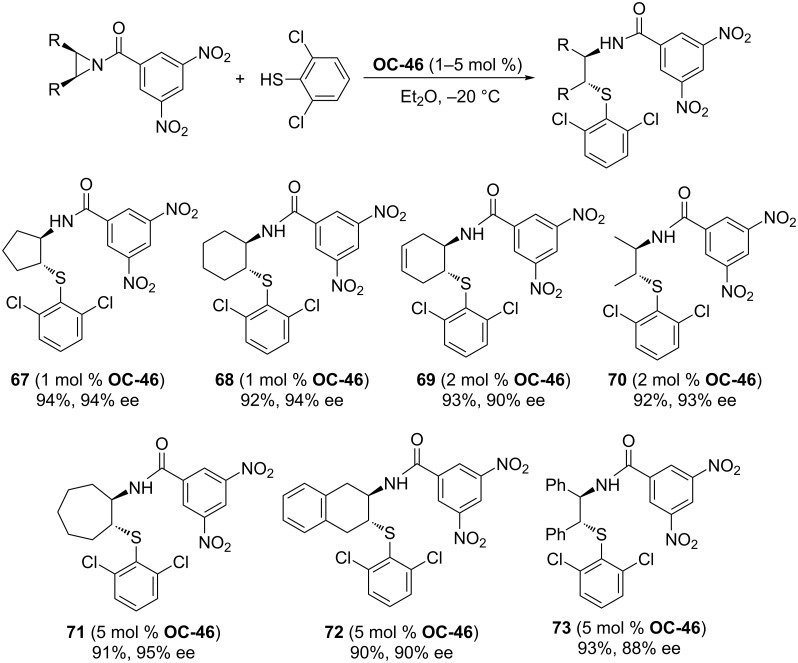
**OC-46** catalyzed desymmetrization of *meso*-aziridines by arylthiols.

**Scheme 10:**
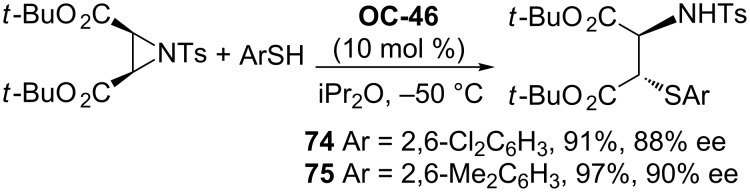
Desymmetrization of *cis*-aziridine-2,3-dicarboxylate.

**Figure 10:**
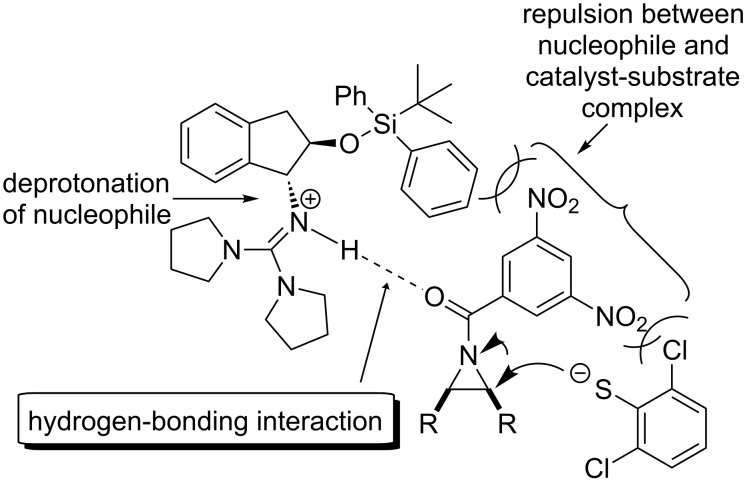
The proposed activation mode of **OC-46**.

**Scheme 11:**
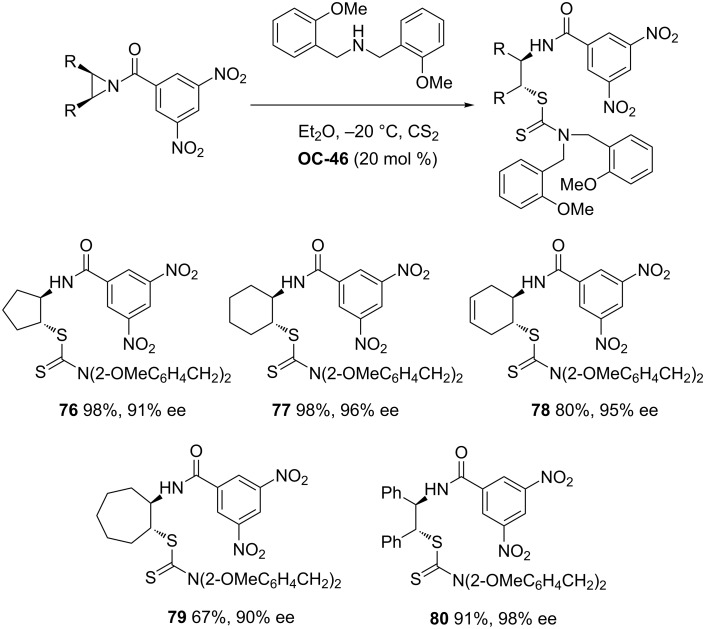
The enantioselective desymmetrization of *meso*-aziridines by amine/CS_2_ in the presence of **OC-46**.

Tan [51] and co-workers developed a series of chiral guanidines based on an amino indanol backbone (Figure 9, **OC-45** to **OC-48**), which proved to be very efficient organocatalysts for the enantioselective desymmetrization of *meso*-aziridines (Scheme 9 and Scheme 10). It was found that *meso*-aziridines and arylthiols containing strong electron-withdrawing groups afforded the desired products with high yields and ee’s. The reaction of *meso*-*N*-3,5-dinitrobenzoylaziridine with 2,6-dichlorobenzenethiol provided the best enantioselectivity of the β-(*N*-acylamino)aryl thioether (up to 92% ee), with only 1 mol % of chiral guanidine **OC-46** as a catalyst. Based on density functional theory (DFT) calculations, a plausible mechanism indicated that the hydrogen-bonding interaction between the chiral guanidine and the carbonyl group in *meso*-*N*-acylaziridine is crucial for the nucleophile attack and the enantioselectivity. It seems that the increase in steric repulsion between nucleophiles and aziridines is helpful to obtain high enantioselectivities of the corresponding products (Figure 10). The desymmetrization of *meso*-aziridines with the in situ generation of carbamodithioic acids from amine and carbon disulfide is also investigated to provide ring-opened products **76** to **80** in high yields and good enantioselectivities (Scheme 11).

#### Chiral 1,2,3-triazolium chlorides

**Figure 11:**
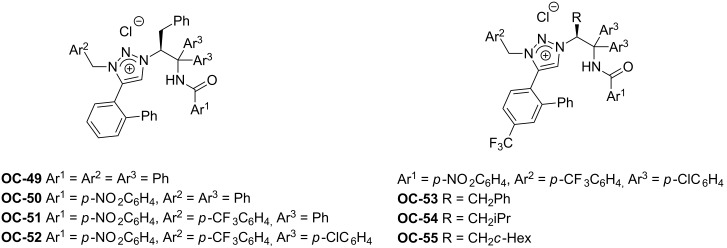
The chiral 1,2,3-triazolium chlorides **OC-49** to **OC-55**.

**Scheme 12:**
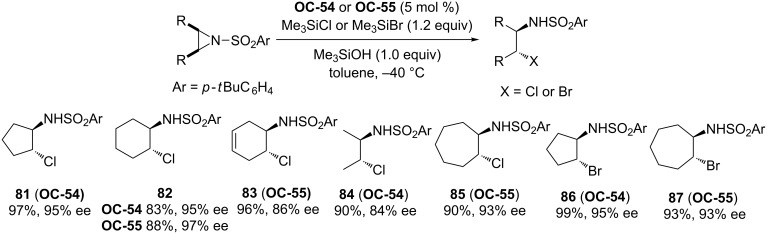
The enantioselective desymmetrization of *meso*-aziridines by Me_3_SiX (X = Cl or Br) in the presence of **OC-54** or **OC-55**.

Most recently, Ooi [52] and colleagues have described a desymmetrization of *meso*-*N*-*p*-*tert*-butylphenylsulfonylaziridines with trimethylsilyl halides by using novel chiral 1,2,3-triazolium chlorides (**OC-49** to **OC-55**) as catalysts (Figure 11). The treatment of *meso*-aziridine with 1 equiv of chiral 1,2,3-triazolium chloride **OC-49**·Cl or Me_3_SiCl in toluene at −40 °C for 12 h did not result in a product formation, but the combined use of a catalytic amount of **OC-49**·Cl (5 mol %) and a stoichiometric quantity of Me_3_SiCl led to the desired ring-opening product in low yield (35%) and moderate enantioselectivity (65% ee). The control experiments indicated that the hypervalent silicate ions were involved in the reaction pathway. The enantioselectivities of the desired products were improved by modifying the substituents of catalysts and additives. It was found that the catalysts of chiral 1,2,3-triazolium chlorides containing electron-withdrawing groups usually afforded the high enantioselectivities. The more sterically hindered groups such as iPr and cyclohexyl in catalysts **OC-54** and **OC-55** demonstrated better enantioselective inductions than the phenyl in **OC-53**. By using 10 mol % of Me_3_SiOH as additive, both high yields (up to 99%) and enantioselectivities (up to 95% ee) of the corresponding β-chloro-*N*-arylsulfonylamines **81** to **87** were obtained (Scheme 12). The additive in the desymmetrization was proposed to facilitate the regeneration of the active chiral triazolium chlorosilicate.

### Organocatalyzed enantioselective desymmetrization of *meso*-epoxides

Compared to aziridines, the epoxides are much more active in their transformations because of the stronger electronegativity of the oxygen atom compared to the nitrogen atom. Some *meso*-epoxides are attacked by various nucleophiles to afford β-functionalized alcohols in the absence of a catalysts [53–60], so the stereo-controlling of the ring-opening of epoxides is quite challenging, and the background reaction should be suppressed to achieve high enantioselectivities. However, the catalytic asymmetric ring-opening of *meso*-epoxides gained much attention by its unique advantages of good atom-economy and versatile transformations. On the one hand, the desymmetrization of *meso*-epoxides has been successfully performed with various nucleophiles in the presence of metal-based chiral Lewis acid catalysts [61–72]. In recent years, expensive and toxic metals were more and more replaced by cheap and environmentally benign elements [73–77] such as Mg, Fe, Ti, Sc and Ni. On the other hand, the enantioselective desymmetrization of *meso*-epoxides by organocatalysts received much less attention, and only a few examples provided the desired products in both high yields and high enantioselectivities. In the presence of some achiral phase-transfer catalysts or phosphines such as TBAB and P(*t*-Bu)_3_, the vicinal chlorohydrins were obtained in high yields. Up to now, three classes of organocatalysts including chiral phosphoramides, chiral phosphine oxides, and chiral pyridine *N*-oxides were used in the enantioselective desymmetrization of *meso*-epoxides.

#### Organocatalyzed enantioselective ring-opening of epoxides in early stage

In 1981, Andrews [78] and co-workers have found that the ring-opening of cyclohexene and cyclopentene oxides with silicon halides was facilitated by the addition of catalytic amounts of tetra-*n*-butylammonium chloride or triphenylphosphine to afford *trans O*-protected vicinal chlorohydrins in quantitative yields. This pioneering work opened the possibility for desymmetrization of *meso*-epoxides by small organic molecules, but the exploration of organocatalysts for the asymmetric ring-opening of epoxides remained stagnant for sixteen years.

**Figure 12:**
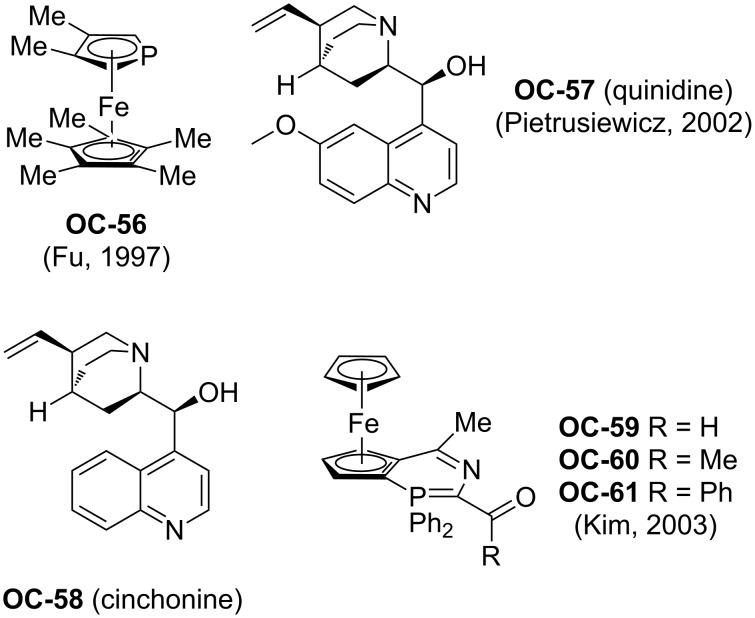
Early organocatalysts for enantioselective desymmetrization of *meso*-epoxides.

In 1997, Fu [79] and colleagues have developed a phosphaferrocene **OC-56** for the organocatalytic ring-opening of epoxides with trimethylsilyl chloride (TMSCl) to provide *trans O*-TMS protected vicinal chlorohydrins in quantitative yields under very mild conditions (Figure 12).

**Scheme 13:**
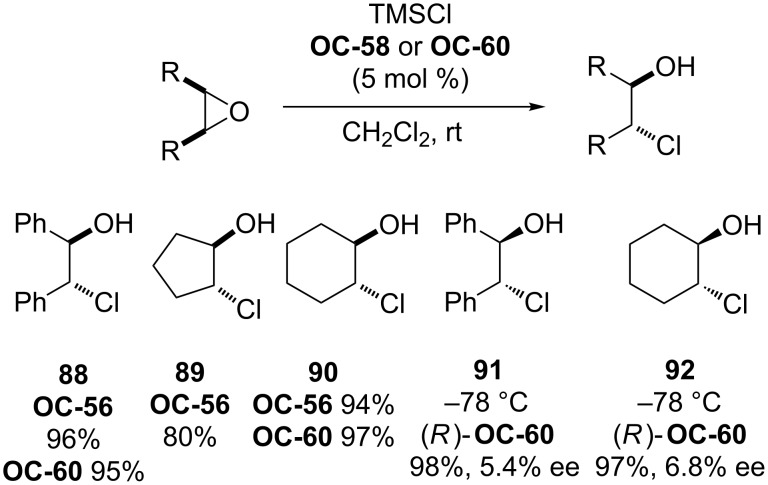
Attempts of enantioselective desymmetrization of *meso*-epoxides in the presence of **OC-58** or **OC-60**.

In 2003, Kim [80] and co-workers have prepared a new class of 1,2-ferrocenediylazaphosphinines (**OC-59** to **OC-61**), which served as organocatalysts for the ring-opening of a series of *meso*-epoxides to produce chlorohydrins with high yields and good regioselectivities. However, in the presence of enantiopure 1,2-ferrocenediylazaphosphinines **OC-59**, the desired chlorohydrins were obtained from corresponding *meso*-epoxides in excellent yields but with quite disappointing enantiomeric excesses (Scheme 13).

**Scheme 14:**
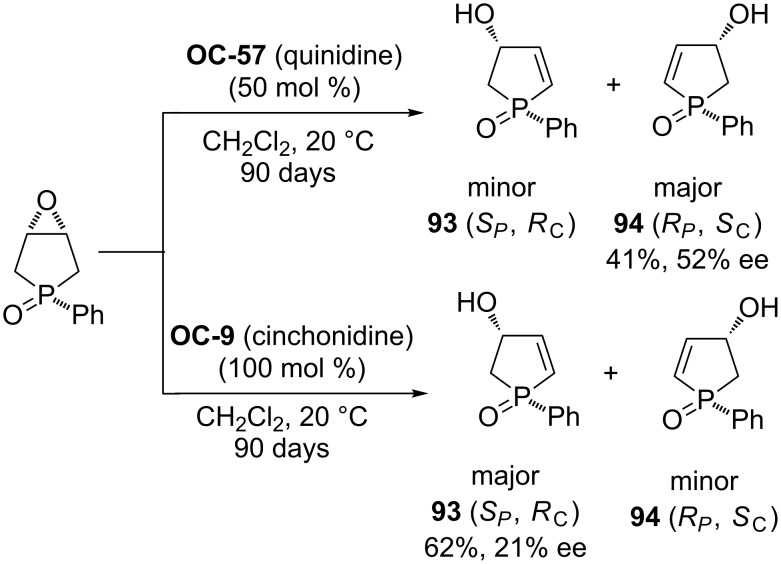
The enantioselective desymmetrization of a *meso*-epoxide containing one *P* atom.

Pietrusiewicz [81] and researchers have discovered the enantioselective desymmetrization of a phospholene meso-epoxide by cinchona alkaloids to *P*,*C*-chirogenic 3-hydroxy-2-phospholene derivatives **93** and **94**. Among the four main components of cinchona alkaloids, quinidine (**OC-57**) proved to be the most effective base in the enantioselective rearrangement of epoxide, and in the presence of 0.5 equiv of quinidine, the corresponding rearranged product **94** was obtained in 41% yield with 52% ee. The other *P*,*C*-chirogenic phospholene derivative **93** was produced by using 100 mol % cinchonidine as a promoter (Scheme 14). But this desymmetrization process is extremely sluggish and the reaction time is up to 90 days!

#### Chiral phosphoramides and phosphine oxides

**Figure 13:**
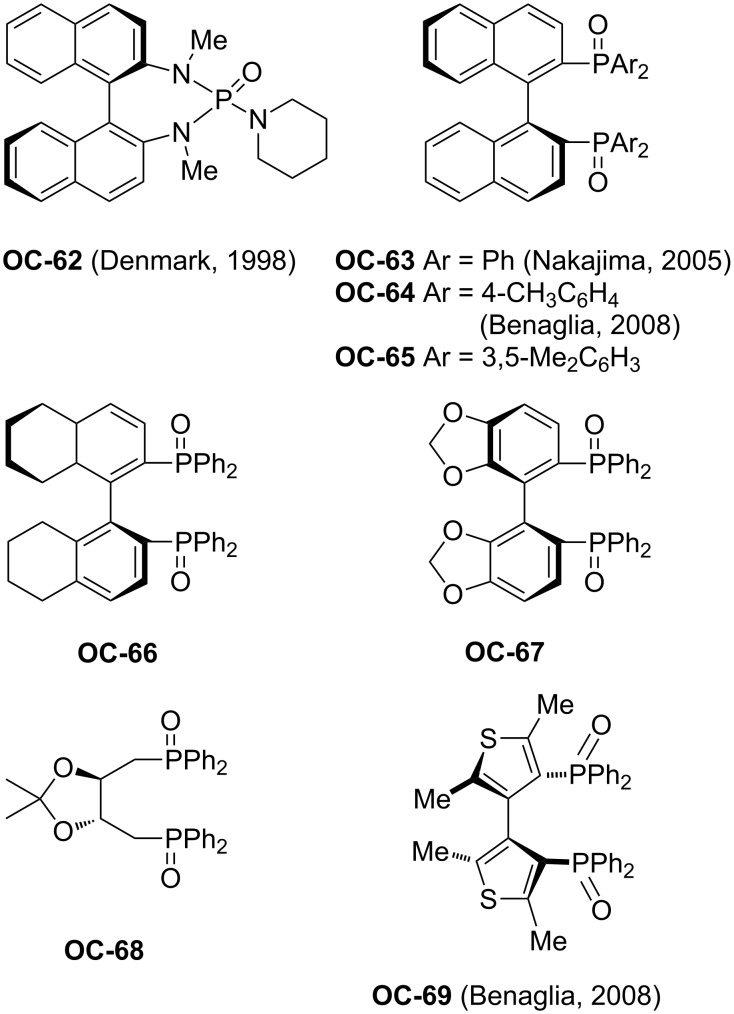
Some chiral phosphoramide and chiral phosphine oxides.

**Scheme 15:**
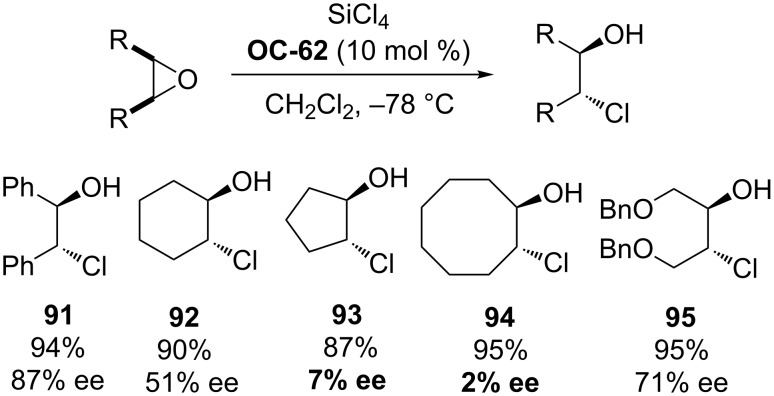
**OC-62** catalyzed enantioselective desymmetrization of *meso*-epoxides by SiCl_4_.

**Figure 14:**
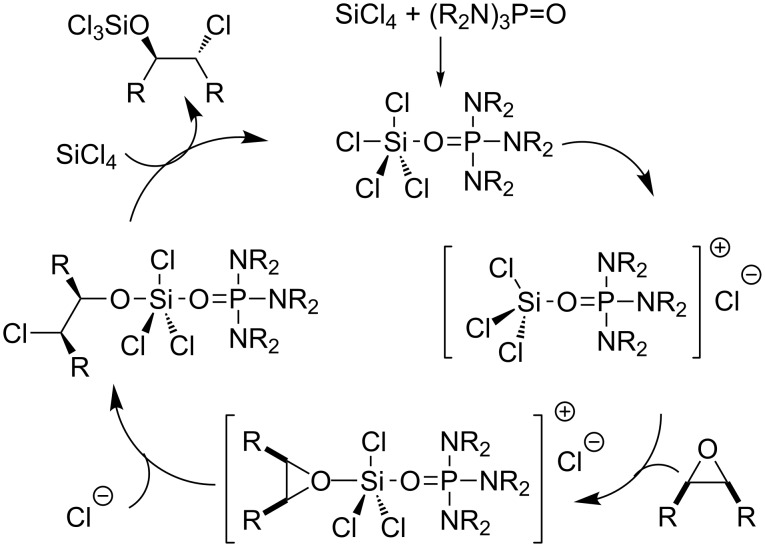
The proposed mechanism of the chiral HMPA-catalyzed desymmetrization of *meso*-epoxides.

The first efficient organocatalyzed enantioselective desymmetrization of *meso*-epoxides was realized by Denmark [82] and colleagues in 1998 (Figure 13). In their initial experiments, various epoxides were attacked with 1.1 equiv of freshly distilled SiCl_4_ by using 10 mol % of HMPA as a catalyst in CH_2_Cl_2_ at −78 °C to afford the corresponding *trans*-vicinal halohydrins in high yields (up to 96%). This exciting discovery provides an opportunity to enantioselectively desymmetrize *meso*-epoxides by chiral phosphoramides. A series of chiral phosphoramides were tested. Among these organocatalysts, the chiral HMPA analogue **OC-62** is most efficient to produce enantiomeric chlorohydrins in good to excellent yields but low enantioselectivities except with acyclic *meso*-epoxides (Scheme 15). The highest enantioselectivity was 87% ee. The proposed reaction mechanism is depicted in Figure 14. The first step of the catalytic cycle is the activation of SiCl_4_ by chiral phosphoramide **OC-62** to form a complex, which was ionized to produce a highly reactive silicon cation and a chloride ion. The epoxide was activated by the chiral complexation of the phosphorus/silicon cation, and then followed by an attack with the chloride ion in an S_N_2 fashion to furnish chlorohydrin enantioselectively (Figure 14).

**Scheme 16:**
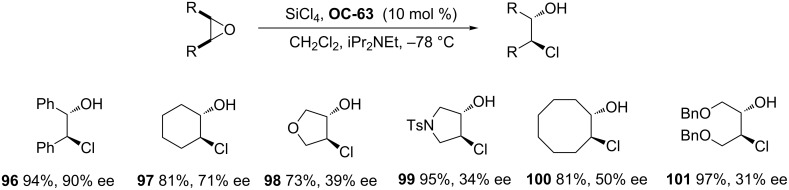
The enantioselective desymmetrization of *meso*-epoxides in the presence of **OC-63**.

From this description, the activation of SiCl_4_ was depending on the formation of the chiral complexation of the phosphorus/silicon cation through the oxygen atom of P=O and the coordination with the silicon atom of the nucleophiles. It can be deduced that the chiral phosphine oxides can also play a role for the activation of SiCl_4_ and serve as catalysts for the desymmetrization of *meso*-epoxides. The first example of a chiral phosphine oxide-catalyzed asymmetric ring-opening of *meso*-epoxides has been realized by Nakajima [83] and co-workers and proved to be very effective. They have prepared a chiral phosphine oxide BINAPO (**OC-63**) based on a binaphthyl-skeleton, which was utilized as an organocatalyst for the enantioselective desymmetrization of *meso*-epoxides by SiCl_4_. In the presence of 1.5 equiv of iPr_2_NEt, various acyclic and cyclic *meso*-epoxides were desymmetrized to enantioenriched chlorohydrins (**96** to **101**) in high yields (Scheme 16). When *meso*-stilbene oxide was used as a substrate, the best enantioselectivity was up to 90% ee under optimum conditions. However, the other substrates, including the *meso*-epoxides derived from butenediol, pyrroline and dihydrofuran, gave quite disappointing enantioselectivities. Interestingly, **OC-65** significantly reduced the enantioselectivities under the optimized conditions, but the chiral phosphine oxides **OC-66** and **OC-67** afforded enantioselectivities similar to that of BINAPO (**OC-63**). This indicated that the substituents on the phenyl ring had a remarkable effect on the enantioselectivities in the desymmetrization of *meso*-epoxides [84]. Benaglia [85] and colleagues have developed chiral phosphine oxides **OC-64** and **OC-69** for the enantioselective desymmetrization of *meso*-stilbene oxide to provide the corresponding vicinal chlorohydrin **96** in up to 82% ee.

**Figure 15:**
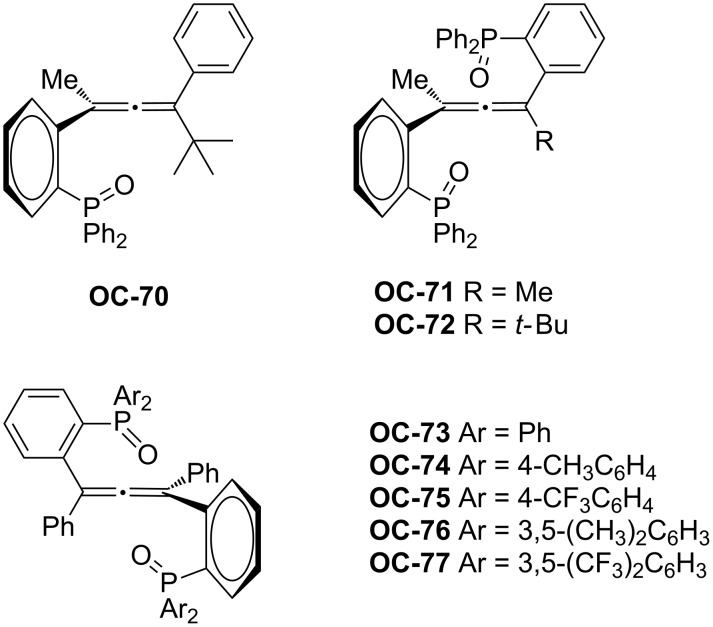
The Chiral phosphine oxides (**OC-70** to **OC-77**) based on an allene backbone.

**Scheme 17:**
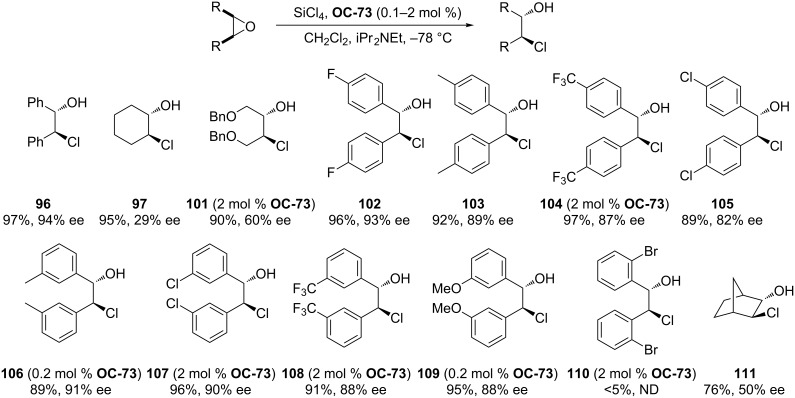
**OC-73** catalyzed enantioselective desymmetrization of *meso*-epoxides by SiCl_4_.

More recently, Ready [86] and co-workers have synthesized a series of chiral phosphine oxides (**OC-70** to **OC-77**) based on an allene backbone (Figure 15). These novel chiral mono- and bisphosphine oxides were investigated as organocatalysts for the efficient enantioselective desymmetrization of *meso*-epoxides. Due to the lack of *C*_2_ symmetry of mono-phosphine oxides, they displayed lower reactivities and enantioselectivities than the bisphosphine oxides. The variation of the substituents on the allene itself was tolerated in the desymmetrization processes, but both the yields and the enantioselectivities are sensitive to changes in the aryl rings on the phosphine oxide. It was found that the diphenyl-substituted catalyst **OC-73** is a highly reactive and enantioselective catalyst to produce the chlorohydrins in up to 94% ee with only 0.1 mol % catalyst-loading. The *cis*-stilbene oxides with substituent in the *meta* or *para* position are tolerated, the yields and enantioselectivities of desired chlorohydrins are satisfying, but *ortho*-substituted stilbene oxides are not reactive in the desymmetrization under the same reaction conditions. Unsatisfactorily, the desymmetrization of linear, cyclic, or bicyclic aliphatic *meso*-epoxides resulted in corresponding chlorohydrins in high yield but with low enantiomeric excesses (Scheme 17).

#### Chiral pyridine *N*-oxides

**Figure 16:**
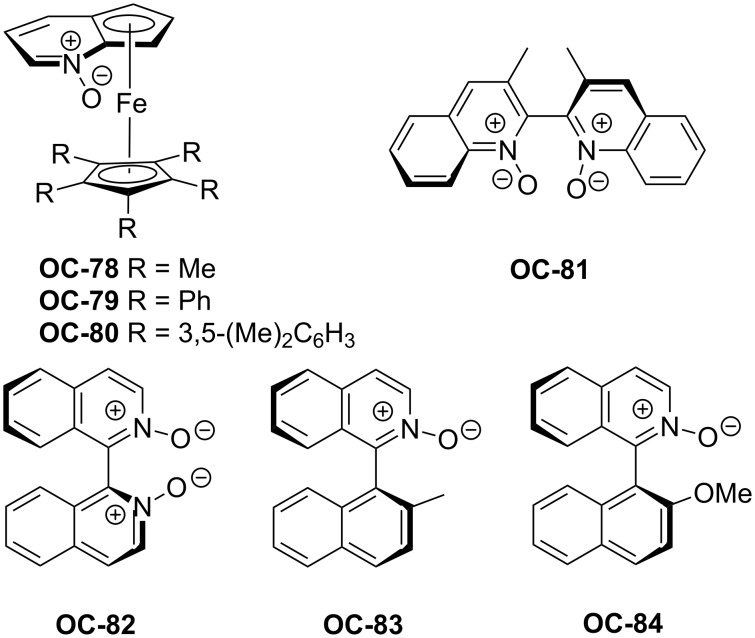
Chiral pyridine *N*-oxides used in enantioselective desymmetrization of *meso*-epoxides.

The breakthrough of the organocatalyzed enantioselective desymmetrization of *meso*-epoxides was the employment of chiral pyridine *N*-oxides as catalysts. In 2001, Fu [87] and colleagues demonstrated the utilization of selected chiral pyridine *N*-oxides in the enantioselective desymmetrization of *meso*-epoxides. These novel chiral pyridine *N*-oxides possess a plane of chirality in a ferrocenyl backbone. The steric hindrance of the Fe(*η*^5^-C_5_Ar_5_) group on catalysts (Figure 16, **OC-78** to **OC-80)** is very crucial to the enantioselectivities of desymmetrization of *meso*-epoxides. The increase of steric hindrance of the Fe(*η*^5^-C_5_Ar_5_) group can indeed lead to increased enantioselectivities of the desired chlorohydrins. For example, by shifting the substituents on Fe(*η*^5^-C_5_Ar_5_) from phenyl (**OC-79**) to 3,5-dimethylphenyl (**OC-80**), the enantioselectivity of desymmetrization of *cis*-stilbene oxide with SiCl_4_ at room-temperature was improved significantly from 25 to 68% ee. The highest enantiomeric excess of chlorohydrin (92% ee) was obtained by simple decreasing the reaction temperature to −78 °C. A series of substituted *cis*-stilbene oxides were desymmetrized in very good yield and high stereoselection, and the substrates with electron-withdrawing group such as F or CF_3_ at the aryl ring can produce chlorohydrins with excellent enantioselectivities (up to 98% ee). Further investigation indicated that the catalyst-loading can be decreased to be 1 mol % and **OC-80** can be recovered nearly quantitatively (>90%) with high ee’s.

**Scheme 18:**
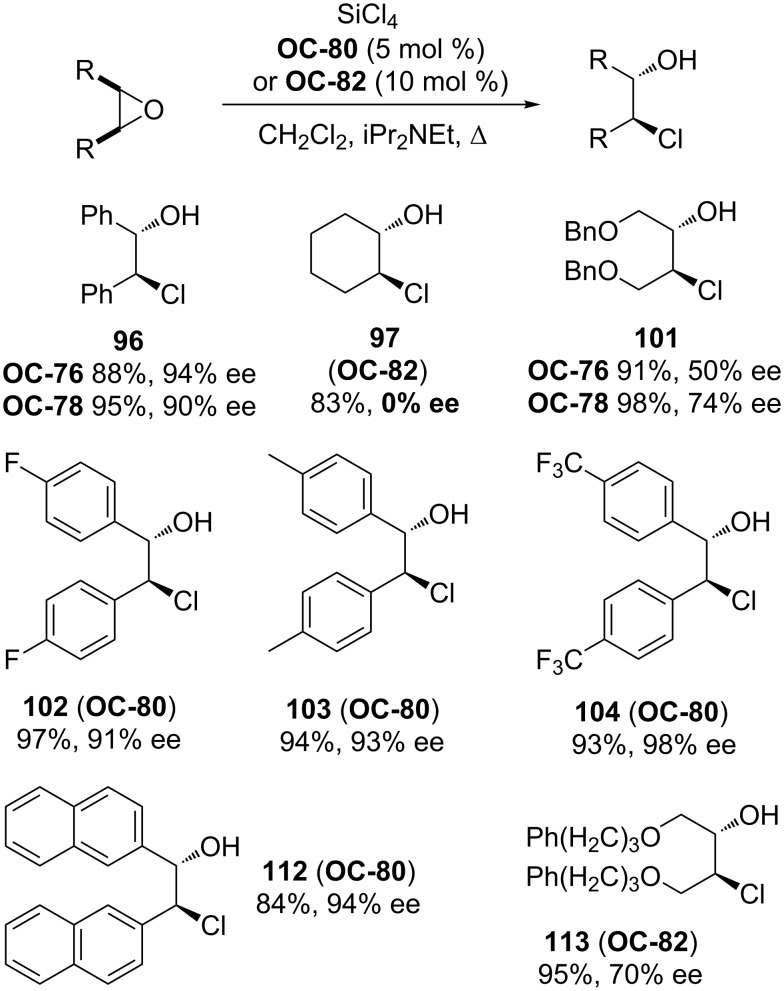
Catalyzed enantioselective desymmetrization of *meso*-epoxides in the presence of **OC-80** or **OC-82**.

After Fu’s work, two axially-chiral bipyridine *N*,*N*’-dioxides **OC-81** and **OC-82** were selected by Nakajima [88] and co-workers for the enantioselective desymmetrization of *meso*-epoxides (Scheme 18). When SiCl_4_ was used as a nucleophile, the desymmetrization of *cis*-stilbene oxide was performed smoothly in the presence of 10 mol % of catalyst **OC-81** in dichloromethane at −78 °C with iPr_2_NEt as a base to give chlorohydrin in 94% yield and 56% ee. The ee of chlorohydrin was increased to 90% by using **OC-82** as a catalyst under the same conditions, but the use of methyl-, allyl-, or phenyltrichlorosilane as a nucleophile gave racemic products. The choice of a proper solvent is very crucial to the stereoselection. For example, THF or toluene as a solvent gave the chlorohydrin in almost racemic form. The substituted *cis*-stilbene oxides can provide the corresponding chlorohydrins in high chemical yields and ee’s, but *meso*-cyclohexene oxide resulted in a racemic product under the optimal conditions. Because of the low catalytic activities of axially-chiral pyridine *N*-oxides **OC-83** or **OC-84** in the desymmetrization process, a hexacoordinate silicate intermediate between SiCl_4_ and chiral bipyridine *N*,*N*’-dioxide **OC-82** was envisaged to explain the good enantioselectivity of catalyst **OC-82**.

**Figure 17:**
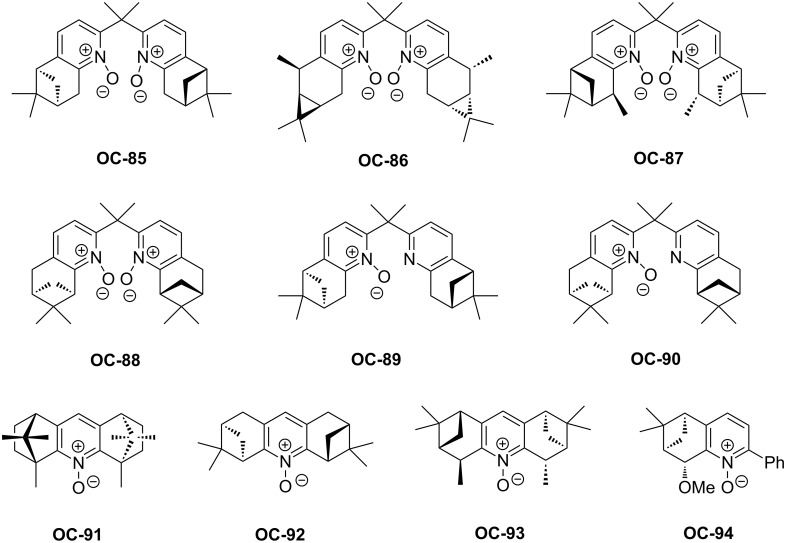
Chiral pyridine *N*-oxides **OC-85** to **OC-94**.

**Scheme 19:**
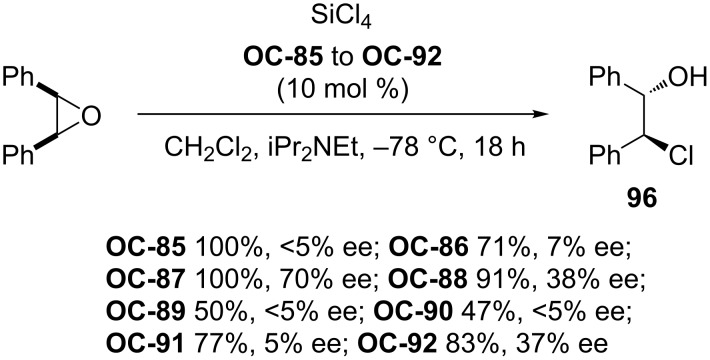
Enantioselective desymmetrization of *cis*-stilbene oxide by using **OC-85** to **OC-92** as catalysts.

In 2008, a set of enantiomerically pure *C*_2_-symmetric bipyridine mono-*N*-oxides and *N*,*N*’-dioxides (**OC-85** to **OC-94**) derived from naturally occurring monoterpenes have been synthesized by Benaglia [89] and colleagues (Figure 17). These chiral bipyridine *N*,*N*’-dioxides were utilized in the enantioselective desymmetrization of *cis*-stilbene oxide and cyclooctene epoxide with SiCl_4_. Some showed high catalytic activities but with low to moderate enantioselectivities. Among them, chiral bipyridine *N*,*N*’-dioxide **OC-87** proved to be the most efficient to produce the corresponding chlorohydrins in quantitative yields and good enantioselectivities (up to 70% ee). Unfortunately, chiral bipyridine mono-*N*-oxides **OC-89** and **OC-90** provided racemic chlorohydrins (Scheme 19).

**Figure 18:**
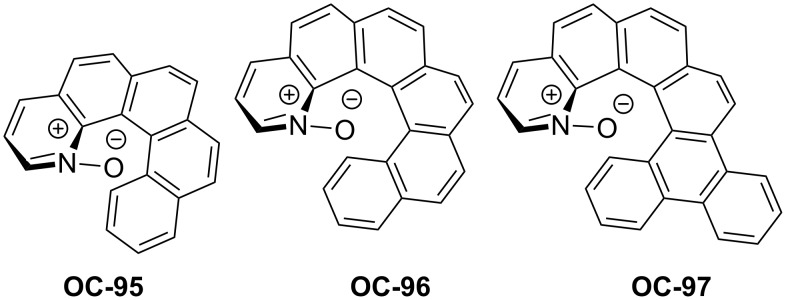
A novel family of helical chiral pyridine *N*-oxides **OC-95** to **OC**-**97**.

**Scheme 20:**
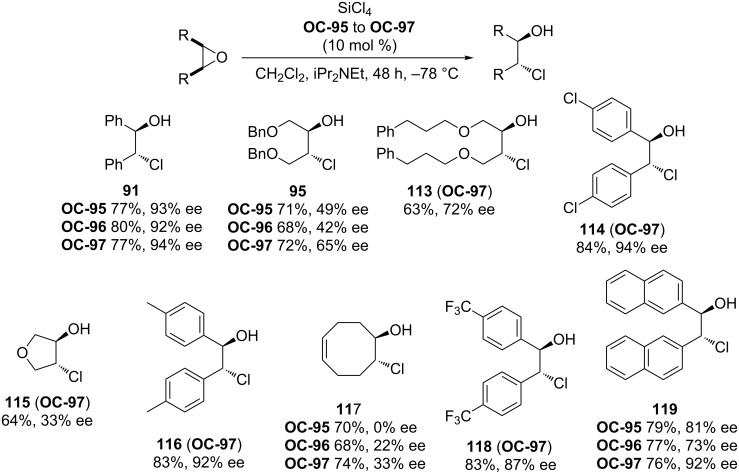
Desymmetrization of *meso*-epoxides catalyzed by **OC-95** to **OC-97**.

Takenaka’s group [90] has developed a novel family of helical chiral pyridine *N*-oxides (Figure 18, **OC-95** to **OC**-**97**) which were employed as catalysts for the enantioselective desymmetrization of *meso*-epoxides with SiCl_4_. All catalysts demonstrated high activities in the desymmetrization process but with different enantioselectivities. The substrates containing aromatic substituents can provide the corresponding chlorohydrins in higher enantioselectivities than those bearing alkyl groups. Unfortunately, the cyclic epoxides produced the chlorohydrins **115** and **117** in low ee’s (Scheme 20).

**Scheme 21:**
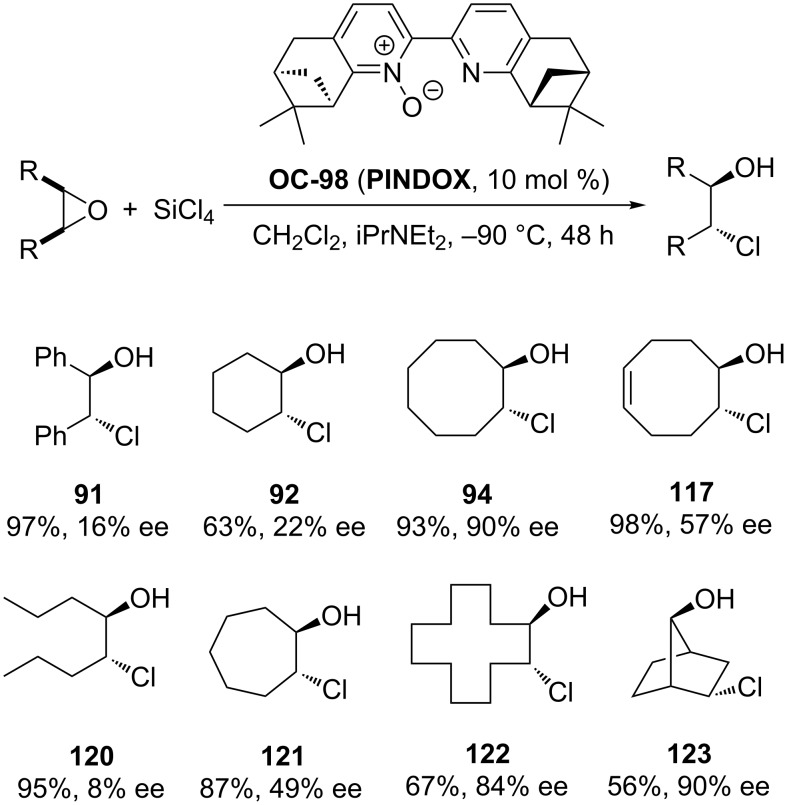
**OC-98** catalyzed enantioselective desymmetrization of *meso*-epoxides by SiCl_4_.

Kočovský [91] and colleagues have introduced a chiral bipyridine mono-*N*-oxide PINDOX **OC-98** for the enantioselective ring-opening of cyclic *meso*-epoxides with SiCl_4_ to produce chlorohydrins in moderate to good enantiomeric excess (Scheme 21). The stereoselectivities are very sensitive to the ring size of the cyclic oxides. For example, when cyclohexene oxide was used as a substrate, the corresponding chlorohydrin was obtained in nearly racemic form. The greater the ring sizes of the cyclic oxides, the higher the enantioselectivities. It was found that the catalyst **OC-98** is unique for the desymmetrization of *meso*-cyclooctene oxide, which was proved to be a very challenging substrate for desymmetrization with other reported organocatalysts, and the chlorohydrin **94** was obtained in 90% ee in the presence of 10 mol % of **OC-98** in dichloromethane at −90 °C. Interestingly, the tricyclic *exo*-norbornene oxide afforded the *syn*-*exo*-chloroalcohol **123** as the major product in 53% yield and 90% ee by a Wagner–Meerwein rearrangement. The mechanism of the enantioselective desymmetrization of *meso*-epoxides by chiral pyridine *N*-oxides is not fully understood at the time of this writing.

## Conclusion

In this review, we summarized the recent advances of the organocatalyzed enantioselective desymmetrization of *meso*-aziridines and *meso*-epoxides. Undoubtedly, these synthetic methodologies will continue to demonstrate their versatile utilities in organic synthesis. However, there are some difficulties that should be pointed out.

Organocatalysts for the enantioselective desymmetrization of *meso*-aziridines are plentiful and can be found in a diverse set of privileged structures, including cinchona alkaloids-based PTCs, *L*-proline-derived amino alcohols, chiral phosphorous acids, chiral thioureas, chiral guanidines, and chiral 1,2,3-triazolium chlorides. But for the desymmetrization of *meso*-epoxides efficient catalysts are presently limited to chiral phosphine oxides and chiral pyridine *N*-oxides. Obviously, there is still a need for the exploration of more efficient organocatalysts with high enantioselectivities in this field. In most cases, the catalyst-loading is up to 10 mol %, and some catalysts are not readily accessible and tunable. Therefore, the search for practical methods for the facile synthesis of organocatalysts, which exhibit a plethora of applications, is still in high demand.

Various C-, N-, S-, Se- and halo-nucleophiles are involved in the organocatalyzed enantioselective desymmetrization of *meso*-aziridines to afford β-functional amine derivatives with high yields and enantioselectivities, while the organocatalyzed enantioselective desymmetrization of *meso*-epoxides is restricted to chloro-nucleophiles. More specifically, SiCl_4_ was used in most cases to produce enantioenriched chlorohydrins. It might be worthwhile to extend research activities toward nucleophiles which are able to produce chiral β-functional alcohols.

The acyclic *meso*-aziridines and *meso*-epoxides usually showed disappointing results except for a few examples. With some further mechanism studies, new and more efficient organocatalysts for the enantioselective desymmetrization of *meso*-aziridines and *meso*-epoxides with a broader substrate scope and milder reaction conditions may be discovered [92–95].
